# Morphological and genetic diversity of camu-camu [*Myrciaria dubia* (Kunth) McVaugh] in the Peruvian Amazon

**DOI:** 10.1371/journal.pone.0179886

**Published:** 2017-06-28

**Authors:** Jan Šmíd, Marie Kalousová, Bohumil Mandák, Jakub Houška, Anna Chládová, Mario Pinedo, Bohdan Lojka

**Affiliations:** 1 Department of Crop Sciences and Agroforestry, Faculty of Tropical AgriSciences, Czech University of Life Sciences, Kamýcká 129, Prague, Czech Republic; 2 Department of Ecology, Faculty of Environmental Sciences, Czech University of Life Sciences, Kamýcká 129, Prague, Czech Republic; 3 Institute of Botany, Academy of Sciences of the Czech Republic, Zámek 1, Průhonice, Czech Republic; 4 Department of Soil Science and Soil Protection, Faculty of Agrobiology, Food and Natural Resources, Czech University of Life Sciences Prague, Prague, Czech Republic; 5 Peruvian Amazon Research Institute. IIAP—PROBOSQUES, Av. Abelardo Quiñones, Iquitos, Peru; National Cheng Kung University, TAIWAN

## Abstract

Camu-camu [*Myrciaria dubia* (Kunth) McVaugh] is currently an important and promising fruit species grown in the Peruvian Amazon, as well as in Brazil, Colombia, and Bolivia. The species is valued for its high content of fruit-based vitamin C. Large plantations have been established only in the last two decades, and a substantial part of the production is still obtained by collecting fruits from the wild. Domestication of the species is at an early stage; most farmers cultivate the plants without any breeding, or only through a simple mass selection process. The main objective of the study was to characterize morphological and genetic variation within and among cultivated and natural populations of camu-camu in the Peruvian Amazon. In total, we sampled 13 populations: ten wild in the Iquitos region, and three cultivated in the Pucallpa region in the Peruvian Amazon. To assess the genetic diversity using seven microsatellite loci, we analyzed samples from ten individual trees per each population (n = 126). Morphological data was collected from five trees from each population (n = 65). The analysis did not reveal statistically significant differences for most of the morphological descriptors. For wild and cultivated populations, the observed heterozygosity was 0.347 and 0.404 (expected 0.516 and 0.506), and the fixation index was 0.328 and 0.200, respectively. Wild populations could be divided into two groups according to the UPGMA and STRUCTURE analysis. In cultivated populations, their approximate origin was determined. Our findings indicate a high genetic diversity among the populations, but also a high degree of inbreeding within the populations. This can be explained by either the isolation of these populations from each other or the low number of individuals in some populations. This high level of genetic diversity can be explored for the selection of superior individuals for further breeding.

## Introduction

The Amazonian tropical ecosystem is characterized by a high biodiversity of plant species (approx. 55,000 species), of which there are more than 150 edible fruit-bearing species used by the local population. However, only a few of these currently have a significant economic importance [1].

Camu-camu [*Myrciaria dubia* (Kunth) McVaugh], a shrub or small tree from the family *Myrtaceae*, has become an economically important fruit species in the recent decades [1]. This species grows naturally in seasonally flooded areas along rivers and oxbow lakes in the Amazonian basin. The economic importance of this species lies in the high content of vitamin C in its cherry-like fruits, which is reported to be in the range from 877 to 3,133 mg per 100 g of pulp. Camu-camu can be considered one of the richest sources of ascorbic acid (vitamin C) of all plant species [2]. Higher ascorbic acid content is present only in the kakadu plum (*Terminalia ferdinandiana* Exell), native to Northern Australia [3, 4] (406–5,320 mg per 100 g of pulp). For centuries, the fruits of the camu-camu have been collected from wild trees, while cultivation is relatively new and plantations established only in the last few decades [5].

There is little information about the genetic diversity of camu-camu. Most of the studies published to date, focusing on this species, mainly targeted the ascorbic acid content of the fruit [6, 7, 8, 9]. However, knowledge about its morphological and genetic diversity is important for further domestication and breeding of new varieties, which can achieve a higher yield, higher vitamin C content, or higher resistance to pests and diseases. For breeding of new varieties, it is necessary to preserve sites with the highest genetic diversity and, thereby, to protect not only the valuable genetic material but also animal species that contribute to its distribution [10, 11]. This knowledge can help us to decide which populations should be protected, how large the protected area should be, and how many plants should be in the population to avoid inbreeding. Knowledge of genetic diversity is therefore important for *in situ* conservation, gene mapping, and finding new lineages. Preservation of genetic material is currently common practice, and defining diversity within and between natural populations is the first step to implementing breeding programs [12].

Due to the large area and specific conditions in which camu-camu grows, genetic diversity would be expected to be high among populations originating in different geographical regions [13, 14]. Here we aimed to characterize the morphological and genetic diversity of cultivated and natural populations of camu-camu in the Peruvian Amazon and compare the genetic diversity among and within these populations. The diversity was assessed by morphological descriptors and genetic analysis using microsatellite markers (SSR markers). Specifically, we asked: (i) is the genetic and morphological diversity high due to the distances between populations and the isolation of individual sites by the Amazonian forest; (ii) are the populations rather inbred due to their small size; and (iii) are the cultivated populations less diverse than wild populations.

## Materials and methods

### Study site and sample collection

**Fig 1:**
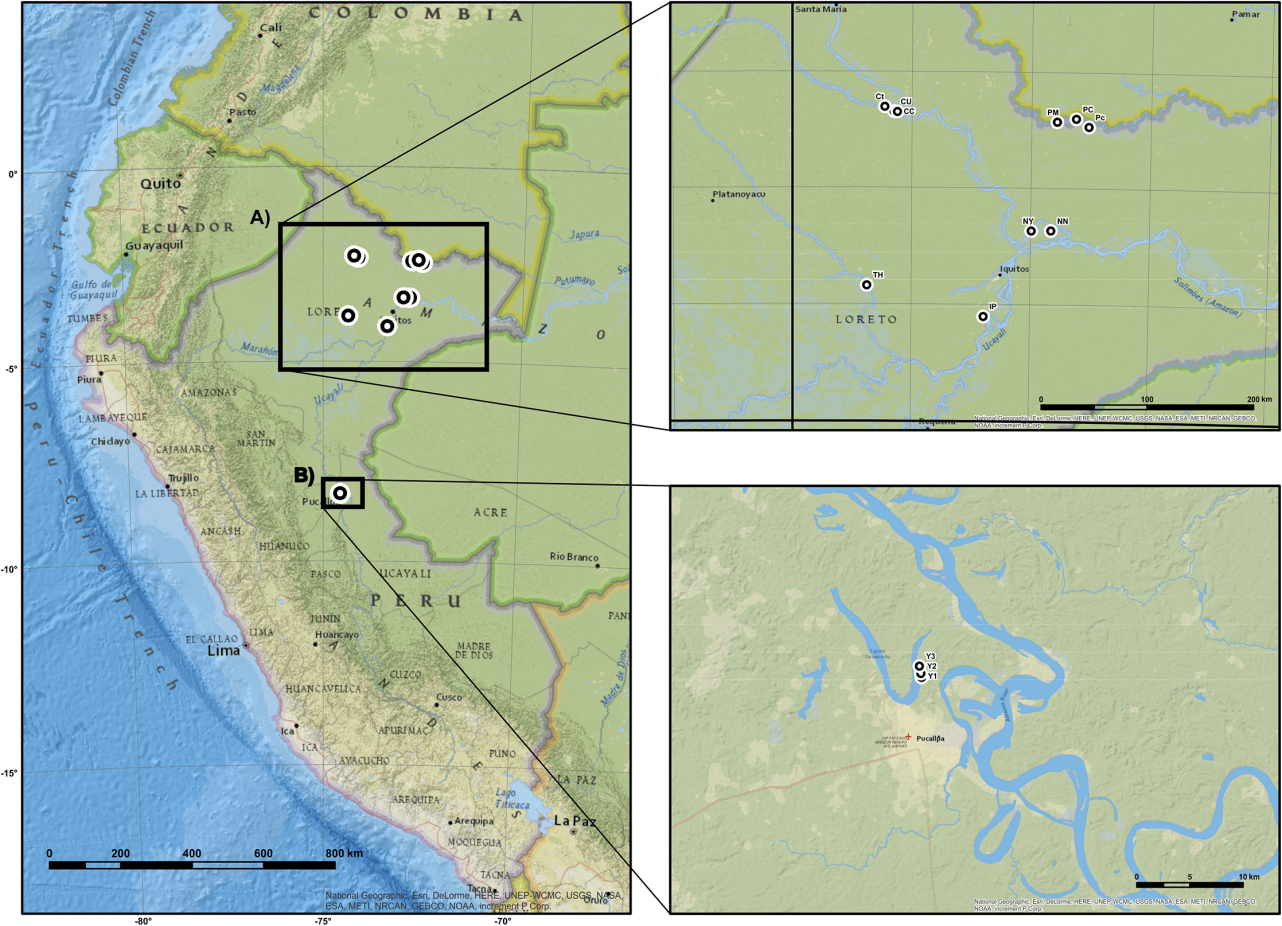
Map of the distribution of *Myrciaria dubia* populations sampled. (A) General distribution of sampled populations. (B) The map represents collected natural populations in Loreto Department. (C) The map represents collected cultivated populations in the area of Pucallpa.

Plant samples were collected between July and September 2015 around the towns of Iquitos and Pucallpa. In Iquitos, plant samples were collected in cooperation with IIAP (Instituto de Investigaciones de la Amazonía Peruana) on their experimental field “San Miguel” (GPS: –3.763404, –73.183840). IIAP is a national Peruvian research institution, and we cooperated with its investigators on the collection of samples in their research collection. From them, we obtained permission to collect samples of leaves for further analysis and to measure morphometric data of selected trees. The experimental field contained a collection of 115 different populations collected from Loreto and Ucayali Departments of Peru. These plants were identical to the parent plants from the natural habitats, as they were directly transplanted from their original environment as young seedlings/saplings (i.e., the plants were propagated by vegetative offshoots and not by seeds). Hence, these populations were considered as natural or wild for the purpose of this study; ten of those populations were chosen for sampling in our study with the aim to cover the largest possible area of natural occurrence of camu-camu in Peru (Fig 1). The (semi)domesticated or, in this study, so-called cultivated populations, were sampled around Pucallpa on three plantations (farms) located near the Yarina Cocha lake (Fig 1), each considered as one population (Table 1). The samples were collected directly from farmer’s fields with the permission of their owners. We encountered no obstacles to our sample collection and morphometric measurements. We did not collect the samples from wild nature. In each population, we randomly selected five individual trees per population for observing major morphological descriptors, and eight to ten trees for leaf tissue sampling. In total, 65 trees from 13 populations were sampled for evaluation of morphological traits. At each individual tree, we randomly selected five leaves, inflorescences and fruits per tree and measured: length/width of the leaves, petiole length, the number of flowers per inflorescence, size and weight of fruits, number and weight of seeds, and the weight of pulp. For genetic analysis, we collected 126 fresh leaf samples (96 from the wild, and 30 from cultivated populations), which were dried by silica gel and stored in plastic bags. We confirm that the field study did not involve endangered or protected species, *M*. *dubia* is a fruit tree widely cultivated in the Amazon region, currently not covered under any protection.

**Table 1:** Location of collected *Myrciaria dubia* populations and summary of genetic diversities within 13 populations of *M*. *dubia*, based on seven microsatellite loci. The table is divided into two parts: Wild (natural populations of camu-camu) and Cultivated (domesticated populations of camu-camu). In the Wild population, the first name refers to the river in which the population is located; the second name is an oxbow lake of that river.

Population	Code	Latitude		Longitude	N (genetics)	N (morph.)	*R*_S_	*H*_o_	*H*_e_	*F*
***Wild***										
Napo- Núñez	NN	-3.36517		-72.828721	10	5	3.000	0.441	0.549	0.206*
Putumayo- Molano	PM	-2.427938		-72.786655	10	5	3.833	0.321	0.573	0.454
Curaray- Urcos	CU	-2.346033		-74.150949	10	5	3.667	0.498	0.629	0.218*
Putumayo- Coto	Pc	-2.46791		-72.520972	10	5	3.167	0.484	0.573	0.164*
Itaya- Pelejo	IP	-4.104278		-73.390674	10	5	2.571	0.286	0.393	0.284*
Curaray- Chavarréa	CC	-2.348431		-74.123527	10	5	3.286	0.276	0.470	0.425*
Putumayo- Cedro	PC	-2.400826		-72.627953	8	5	4.600	0.510	0.633	0.205*
Napo- Yuracyacu	NY	-3.366996		-72.992825	10	5	3.714	0.389	0.556	0.312
Tigre- Huacamayo	TH	-3.840597		-74.37356	10	5	3.429	0.386	0.563	0.327*
Curaray- Tostado	Ct	-2.303415		-74.228746	10	5	3.143	0.281	0.487	0.442*
**Wild total**					98	50	2.965	0.347	0.516	0.328
***Cultivated***										
Yarina Cocha 1	Y1	-8.328365		-74.562932	8	5	3.667	0.327	0.518	0.384*
Yarina Cocha 2	Y2	-8.324925		-74.563511	10	5	3.286	0.495	0.551	0.107
Yarina Cocha 3	Y3	-8.318810		-74.564756	10	5	3.286	0.325	0.553	0.425*
**Cultivated total**					28	15	2.997	0.404	0.506	0.200
**All samples**					**126**	**65**				

**Code—**population designation; **N**- the number of sampled trees; **R**_**S**_**—**allelic richness; **H**_o_- observed heterozygosity; **H**_**e**_- expected heterozygosity; **F**- fixation index, Populations deviating from the Hardy-Weinberg equilibrium at P < 0.05 are marked with an asterisk.

#### DNA isolation

DNA was extracted from the dried leaves using a modified CTAB method [15]. Before the extraction, the dried leaves were homogenized by a crusher machine. Approximately 0.1 g of dried leaf was used. We then progressed to the second phase of the CTAB method [16]. Homogenized plant material was added to the tubes with 500 μl of CTAB buffer and 10 μl of mercaptoethanol. Samples were incubated for 30 min at 60°C. Next, 500 μl of chloroform isoamylalcohol (24:1) was added, and samples were left at room temperature for 10 min. Then, they were centrifuged for 15 min at 9,000 rpm, and the supernatant was extracted into new tubes. Isopropanol (0.7-times the volume of the supernatant) was added to the supernatant and the tubes were left in the freezer for 1 h at –18°C. Samples were then centrifuged for 15 min at 14,000 rpm, the supernatant was discarded, and 500 μl of 70% ethanol was added. The samples were centrifuged for 15 min at 14,000 rpm, the supernatant was removed, and the DNA pellet was dried at room temperature. The pellet was dissolved in 100 μl of TE buffer with 5 μl of RNase, the tubes were briefly mixed, and the content and purity of DNA were measured on a Nanodrop (Thermo Scientific) spectrophotometer.

#### Microsatellite analysis

In the GenBank database [17], several accession of *M*. *dubia* DNA sequences were identified [MDI003 (EX151484.1); MDI004 (EX151485.1); MDI006 (EX151486.1); MDI007 (EX151487.1); MDI009 (EX151488.1); MDI010 (EX151489.1); MDI015 (EX151491.1)] and, based on these sequences, seven specific microsatellite primer pairs were designed (Table 2). For the calculation of melting temperature, the Oligo Analyzer 3.1 from the IDT® Company was used. The first multiplex (M1) composed of the primers MDI015, MDI010, MDI006, and MDI004 were ordered from Generi Biotech and labeled with blue color (FAM). The second multiplex (M2) composed of the primers labeled with different colors MDI009 (VIC), MDI007 (NED) and MDI003 (PET), ordered from Thermo Fisher. The optimal annealing temperature was determined to be 51°C for M1 and 57°C for M2. Next, PCR reactions for M1 were performed with modified volumes of each primer (MDI010, 0.1 μl; MDI006, 0.3 μl; MDI015, 0.6 μl; and MDI004, 1 μl). The PCR reaction mixtures consisted of 1 μl containing 5–40 ng of the genomic DNA template of each sample, primers in the ratio described above (10 μM), 1.2 μl of 5× Colourless GoTaq® Flexi Buffer (Promega, USA), 0.24 μl of 10 mM nucleotides, 1.2 μl of 25 mM MgCl2 (Promega, USA), 0.12 μl 5U GoTaq®Flexi DNA Polymerase (Promega, USA), and 6.24 μl of nuclease free water, for a total volume of 11 μl.

**Table 2:** Microsatellite primers newly designed for *Myrciaria dubia* and used in this study.

Microsatellite GenBank accession no.	5‘ Forward primer	Ta(°C)	Size (bp)	Motif	k	*H*_O_	*H*_E_	*HW*	*F*	*R*_S_	B
	3‘ Reverse primer										
**MDI003 EX151484.1**	GCATAAATAACCCCGCGGTCTC GTACAGCTCCCAGCAGGAGT	59	156	(CT)6(AG)14	9	0.408	0.680	0.00020	0.390	3.58	0.0228
**MDI004 EX151485.1**	GCCTTCCAGACCCTTTTGC GTTCTTGAACCGGGACGC	56	397	(CTT)10	3	0.137	0.218	0.00020	0.390	1.86	0.0415
**MDI006 EX151486.1**	GCTCTCTCTCTGAGTACCTGAAAC CTTTCACGCAAGACCGACG	56	195	(CT)5(CT)6 (CT)12(TCG)11	8	0.421	0.626	0.00020	0.317	3.59	0.0335
**MDI007 EX151487.1**	TCTCGAGAGCTTTCCTCGGAG AGTACTTCACTCTGTCCGGCC	58	245	(CTT)10	4	0.284	0.397	0.00020	0.298	2.48	0.1489
**MDI009 EX151488.1**	CGAAGTCCTGACCTGTTCTGAGTT GCAGACCAGCGAGTTTACACC	59	363	(GA)18	10	0.426	0.661	0.00020	0.351	4.19	0.5330
**MDI010 EX151489.1**	CGATCGCTGCCCTTTCTG GGTTCGGGAGGGTAGGAG	56	95	(CTT)12	3	0.296	0.354	0.06160	0.160	2.04	0.0000
**MDI015 EX151491.1**	CTGTACCTGCATCGATGGTG CGTTCTAATCCGCCATTATTCGTC	56	305	(TC)12	4	0.527	0.647	0.00160	0.186	3.46	0.0274

**k**: number of detected alleles at the locus; **H**_**o**_: observed heterozygosity; **H**_**e**_: expected heterozygosity; ***HW***: significance of deviation from Hardy-Weinberg equilibrium; **F**: fixation index; **R**_**S**:_ allelic richness; **B**: null allele frequency averaged over all populations (Brookfield method)

In the PCR reaction of M2, 0.5 μl of each primer was added to the total volume 10.5 μl. PCR amplifications were performed with the Thermal Cycler T 100 (Bio-Rad, USA) with the following profile: 95°C for 2 min; followed by 30 cycles of 95°C for 1 min, either 51°C (M1) or 57°C (M2) for 90 s, 72°C for 1 min; followed by a hold at 72°C for 5 min. The PCR products were separated by electrophoresis in an ABI PRISM 3500 sequencer (Applied Biosystems, USA). A 1-μL aliquot of PCR product was mixed with 0.5 μL of GeneScan-500 LIZ (Applied Biosystems) and 12 μL of Hi–Di formamide (Applied Biosystems). Allele sizes were determined using GeneMarker version 2.4.0 (SoftGenetics, USA). A microsatellite locus was treated as missing data after two or more amplification failures. Null allele frequencies were calculated using the Brookfield 1 equation [18, 19], and no null allele was presented.

### Data analysis

To evaluate morphological data we used several statistical methods. Because the sampled trees of each population were selected randomly across the experimental orchard, and five leaves/inflorescences/fruits were sampled per tree, and to avoid the strong effect of randomness in sampling morphologically highly variable parameters within one single tree, we used the linear mixed-effect model to assess differences between wild and cultivated populations. The data was evaluated in R software using a linear mixed-effects model (R, [nlme] package, *lme* command, with nested design—random effect *tree*), Principal Component Analysis (R, [stats] package, *prcomp* command). PCA diagrams were visualized using (R, [devtools] and [ggbiplot] packages, *ggbiplot* command) [20].

To evaluate the genetic data, summary data for SSR loci, including the mean allelic richness (*R*_S_) (here allelic richness is a metric that uses a rarefaction index to take into account differences in sample size) [21, 22], and Weir & Cockerham’s parameter *f*(F) (a measure of deviation from random mating within a population) [23] were calculated using Fstat 1.2 [21]. Observed (H_O_) and expected (H_E_) heterozygosities were calculated using Arlequin [24], and deviation from the Hardy-Weinberg equilibrium was determined based on 10,000 permutations in Fstat.

Euclidean distances among all samples were also employed to obtain an unweighted pair group method with an arithmetic mean (UPGMA) phenogram (calculated using Past–[25]).

In STRUCTURE software, the number of genetic clusters (*K*) was estimated, and individuals were fractionally assigned to the inferred clusters. We applied a model, which allows population admixture and correlated allele frequency [26]. Ten replicates for each *K* = 2–6 (the user-defined number of clusters) were set up to confirm the repeatability of the results. Each run comprised a burn-in period of 25,000 iterations, followed by 100,000 Markov chain Monte Carlo (MCMC) steps. The STRUCTURE output data was parsed using the Structure-sum script in R [27], mainly to determine the optimal *K* value following the method of Nordborg et al. [28] and Evanno et al. [29]. Alignment of cluster assignments across replicates analyses was then conducted in CLUMPP 1.1.2 [30] and subsequently visualized using DISTRUCT 1.1 [31].

## Results

### Morphological diversity

**Fig 2:**
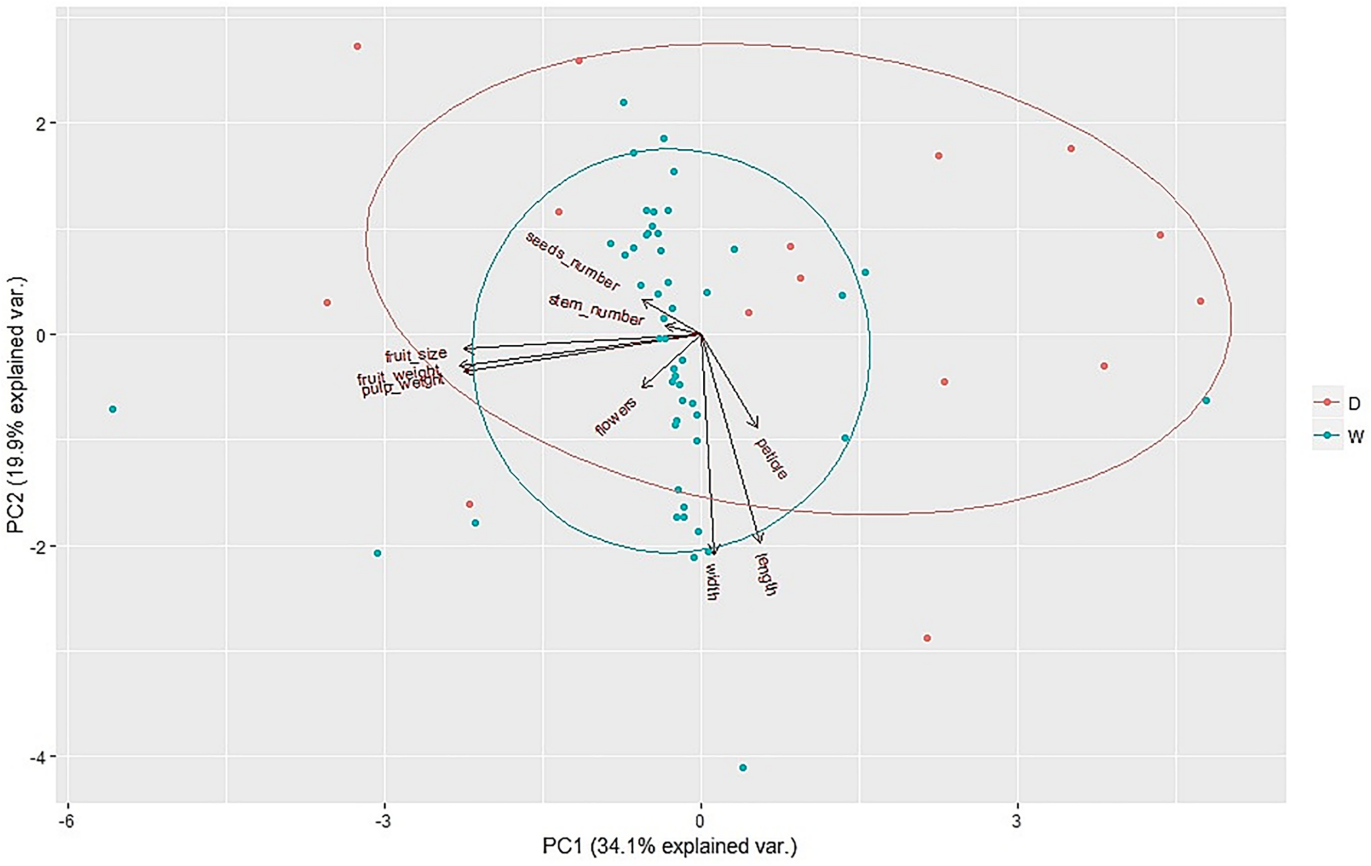
Principal component analysis of morphological parameters. Comparison of Wild versus Domesticated populations. Points represent individual trees; arrows individual parameters; red dots/oval cultivated populations and blue dots/oval wild populations.

Morphological data was calculated using Analysis of Variance for the linear mixed-effects model (ANOVA) (Table 3) and Principal Component Analysis (PCA) (Fig 2).

**Table 3:** Linear mixed-effects models comparing wild and domesticated populations of *Myrciaria dubia*.

Parameter	Wild/Domesticated							
	numDF	denDF	F-value	p-value	median	SD	median	SD
**Length of the leaf**	1	63	0.095	0.759	9.21	0.95	9.07	0.79
**Width of the leaf**	1	63	2.043	0.158	3.37	0.43	3.19	0.39
**Length/width ratio**	1	63	2.517	0.118	2.72*	0.33	2.89*	0.32
**Petiole length**	1	63	0.894	0.348	0.63	0.22	0.67	0.42
**Number of flowers**	1	29	0.585	0.451	4.00	1.21	3.57	1.16
**Size of fruit**	1	21	0.325	0.575	2.59	0.24	2.54	0.19
**Weight of fruit**	1	21	1.239	0.283	10.82	2.70*	9.75	1.74*
**Weight of seeds**	1	21	0.923	0.348	2.31	0.74	2.74	0.66
**Weight of pulp**	1	21	3.132	0.091	8.54	2.15*	7.05	1.23*
**Number of seeds**	1	21	4.454*	0.047*	2.67	0.81	3.00	0.70

**NumDF**: degrees of freedom in the numerator, **denDF**: degrees of freedom in the denominator; **SD**: standard deviation.

* Data was found to be significantly different at p ≤ 0.05 between wild and cultivated population

Using ANOVA, we detected no differences in the morphological data, except in the number of seeds. The number of seeds was higher in the wild populations. PCA analysis is congruent with ANOVA, and only confirmed the morphological similarity between wild and cultivated populations (Fig 2, S1 Table).

### Genetic diversity

In total, 126 samples from 13 populations were analyzed using seven polymorphic SSR primers. All microsatellite loci were polymorphic, with 91 alleles identified (Table 1). The average number of alleles per locus was 3.4 ± 1.6. The value of allelic richness ranged from 1 to 6.5 with an average of 3.0 ± 1.2. Observed heterozygosity (H_o_) varied from 0.137 to 0.527 with an average of 0.357 ± 0.128. Expected heterozygosity (H_E_) varied from 0.218 to 0.680 with an average of 0.512 ± 0.185. Heterozygote deficit was significant in the majority of populations with a high fixation index [*f*(F) = 0.304 ± 0.117] (Table 1). Differences in allelic richness, fixation index, fixation index, and observed *vs*. expected heterozygosity were not significant between wild and cultivated populations (S2 Table,).

**Fig 3:**
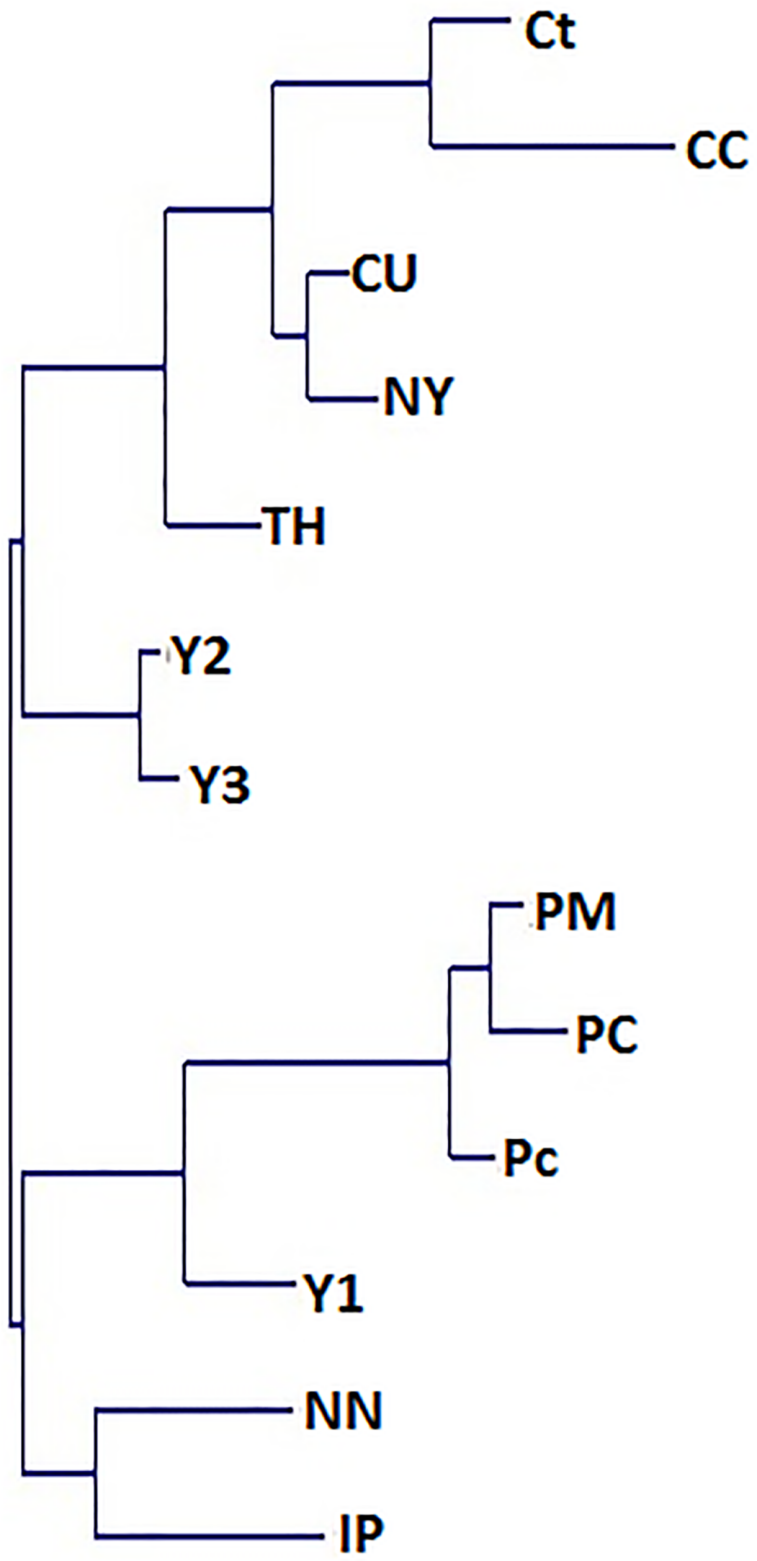
Unweighted pair group method with arithmetic mean phenogram, constructed by seven microsatellite loci using Euclidian distances for 13 populations of *Myrciaria dubia* in Peru. Curaray (CC, Ct, CU); Itaya (IP); Napo (NN, NY); Putumayo (PC, Pc, PM); Tigre (TH); Yarina cocha (Y1, Y2 and Y3) (for population locations, see Fig 1).

By UPGMA analysis, we found that the populations could be divided into two distinct groups and that these groups could each be further subdivided into two subgroups (Fig 3). The division of the cultivated populations is interesting. According to our findings, the Y1 population belongs to the group of populations from the Putumayo River. Whereas the Y2 and Y3 populations were assigned to the populations from the rivers Curaray (CU, Ct, CC), Tigre (TH), and Napo (NY). Populations from the rivers Napo (NN) and Itaya (IP) were surprisingly assigned to the group of populations from the Putumayo River, though geographically very distant.

**Fig 4:**
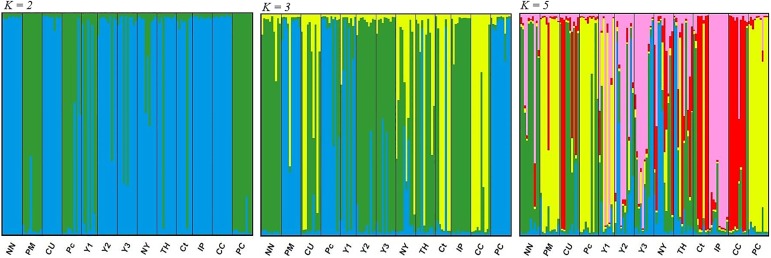
Populations divided into two (K = 2), three (K = 3), and five (K = 5) clusters from STRUCTURE software.

The same division of populations was determined by PCoA (Principal Coordinate Analysis) (S1 Fig) and STRUCTURE analysis. While the analysis of similarity coefficients (S2 Fig) indicated that two, three, and five clusters best explained the genetic structuring of *M*. *dubia* populations by STRUCTURE (Fig 4), Δ*K* (S3 Fig) indicated that only two clusters are informative.

**Fig 5:**
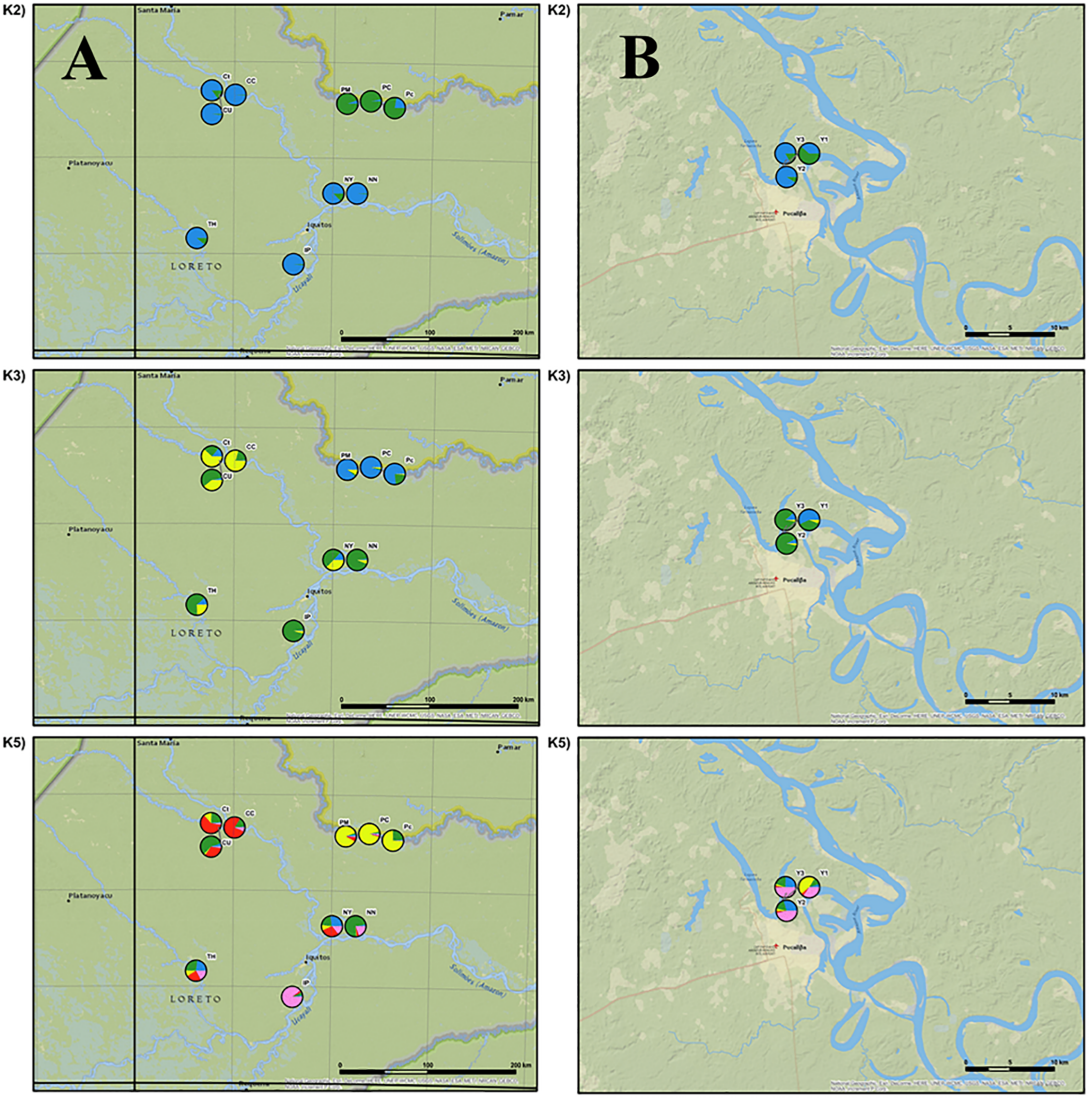
(A) Division of wild populations into two (K = 2), three (K = 3), and five (K = 5) clusters inferred by STRUCTURE and graphically processed into the map. (B) Division of cultivated populations into two (K = 2), three (K = 3), and five (K = 5) clusters inferred by STRUCTURE and graphically processed into the map.

The genetic cluster (*K* = 2), depicted by blue color, is dominated by populations from the Curaray (CC, Ct, and CU), Tigre (TH), Itaya (IP), and Napo (NN, NY) rivers, while populations PC, Pc, and PM are from the Putumayo River (green color). The cultivated populations from Pucallpa (Y2 and Y3) were assigned to the first genetic cluster (the Curaray, Tigre, Napo and Itaya rivers) and population Y1 is genetically closely related to the populations from the Putumayo River (Fig 5).

When the populations were divided into three genetic clusters (*K* = 3), populations from the Putumayo River (PM, Pc, and PC) formed a separate genetic cluster. The rest of the populations were divided according to the river system into two groups (i.e. the Curaray River, with the prevailing cluster depicted in yellow, and the Napo, Tigre and Itaya rivers shown in green). The genetic composition of the cultivated populations showed that they were combined from only two areas (i.e., from the Putumayo River and the Napo, Tigre, or Itaya rivers) (Fig 5).

By division into five genetic clusters, we were able to recognize four geographically distinct groups of populations along different rivers, especially when populations occurring in the Napo, Tigre and Itaya rivers were further divided. The cultivated populations Y2 and Y3 were assigned to the population Itaya–Pelejo. Cultivated population Y1 remains part of the “Putumayo” group (Fig 5).

## Discussion

### Morphological diversity

The measured morphological characteristics showed low variability within and among populations. The wild and cultivated populations were not found to be significantly different, and we did not detect any differences among the populations. The only significant difference we detected was the number of seeds per fruit, where the cultivated populations had a higher number of seeds per fruit. These results clearly show a low degree of domestication of *M*. *dubia* in the Peruvian Amazon. It is evident those plants grown in plantations were neither selected nor bred, and that they possess quite similar phenotypical characteristics to wild plants. The first attempt at the cultivation of camu-camu in Peru was in 1980, and this trial was established near Iquitos [32]. Thus, the domestication process started only about four decades ago.

According to our results, the use of morphological descriptors for quantitative and qualitative traits of leaves had only little significance. Greater importance should be placed on the detection of fruit characteristics such as diameter, weight, and number of seeds, the weight of the pulp, and vitamin C content, which was not included in this study. It seems that cultivated populations of camu-camu were not yet highly selected according to fruit morphological traits, and, seemingly, there remains potential to select individuals with larger fruits from wild populations.

### Genetic diversity

We are aware of the limitation of our study as the sample size per population was relatively low (8–10 trees per population), so that the present results might be different if the sample size of the populations was increased. This is because the estimated genetic diversity indices are strongly dependent of the accurate estimate of gene frequencies within populations.

However, compared to our morphological characteristics findings, the microsatellite loci detected a high level of genetic diversity among and within populations. The expected heterozygosity was higher than the observed heterozygosity, and the fixation index showed high rates. Similar results were also obtained by Rojas et al. [14] and Koshikene [13], who also used microsatellite markers. In both cases, the values of expected and observed heterozygosity of wild populations were higher than in our study. For wild camu-camu populations in Brazil, Rojas et al. [14] found that the average value of expected and observed heterozygosity were 0.797 and 0.409 respectively. The average value of the fixation index was 0.377, and the average number of alleles across all loci was 12.7. Slightly lower values were determined by Koshikene [13] in Brazil. The lower values of heterozygosity and coefficient of inbreeding reached in our study can be explained by the smaller geographical area from which the samples were collected. In the two previous studies, the samples were collected in areas that covered the whole Amazonian region of Brazil. Despite the high diversity measured in our study (and the previous two studies), the fixation index was high. In all three studies, the results can be explained in several ways. Some possible explanations of high genetic diversity are the relatively large distances between populations, and that the populations located along main rivers are isolated from each other by upland tropical rainforest, which forms a barrier the species cannot cross. Migration of the species through the upland forest is less probable because the seeds are mainly dispersed by water and fish. Oxbow lakes, where trees grow naturally, are relatively small, and the populations are dense. According to Rojas et al. [14], outcrossing and low gene flow took place, resulting in a higher fixation index. The bottleneck effect might also account for our findings in some populations. During some unexpected events, the number of individuals in the population is reduced, so that many alleles are lost when the population is restored [33].

Franceschinelli et al. [34] performed similar research on *Myrciaria floribunda*, which is native to the wet forests of Central and South America. Allozyme markers were used to determine the genetic diversity. The fixation index was 0.153, which is lower than that reported here for *M*. *dubia*. This is likely because allozyme markers are less variable than SSR markers. Nevertheless, allozyme markers were still able to demonstrate higher values of homozygotes in the populations compared to our study. The higher fixation index was again explained by the bottleneck effect and low gene flow, and this confirms the results of our study.

In the dendrogram, it can be seen that populations were divided into groups depending on the river watershed where they were located. For example, in our case, population Napo-Núñez (NN) is located close to the conflux of the Napo and Amazon rivers. Population Itaya–Pelejo (IP) was located on the Itaya River, which also flows into the Amazon, and this conflux is not far from the conflux of the Napo and Amazon. During the rainy season, some fruits were probably naturally transported from the Itaya River to the tree populations of the Napo River, and genes were mixed. This can be a possible explanation why populations NN and IP belong to the same group, and why population NN is not connected with population NY from the same river. This theory was supported by Rojas et al. [14], who achieved similar results.

STRUCTURE software divided the natural populations into two distinct groups. Populations PM, Pc, and PC make up the first group, lying on the river Putumayo. This river is geographically remote from other populations, and upland tropical rainforest complicates its connection with those populations. This is because the seeds of *M*. *dubia* are primarily distributed by water during floods, and pollen is not transmitted over longer distances. Populations from the second group are closer in proximity, and the rivers are often connected during floods and, thus, enable the transfer of genetic material.

The cultivated populations can also be divided into two groups according to their origin. The populations Y2 and Y3 originated in the catchment area of the rivers Curaray, Tigre, Napo, and Itaya, and were brought by farmers to the Pucallpa region. STRUCTURE software (K = 5) assigned the origins of these populations closer to Iquitos city, around the river Itaya. The Y1 population was assigned to the other group, and its origin lies in the area of the river Putumayo on the border between Peru and Colombia, flowing into Brazil. Further research will be necessary to confirm this hypothesis.

Genetic analysis revealed significant differences between wild populations. These observed differences in genetic diversity should be used for the preservation of wild genotypes *in situ* and *ex situ*, and the most promising genotypes should be stored in germplasm banks. For further studies focused on this species, we propose to also collect plant material from the peripheral areas of its occurrence (Ecuador, Bolivia, Venezuela, and Guyana). Plants from those marginal areas might be more resistant to unfavorable climatic conditions and could allow the cultivation of this species outside of the area of its natural occurrence. It is also recommendable to compare the variability of populations from the Peruvian Amazon and Brazil, to find populations with superior plants for further breeding. The fruit collection from wild populations leads to losses of genetic material because the excessive collection of the fruits reduces the amount of seeds, and thus might reduce the number of plants in the population. These losses might affect the structure of these populations in the future and cause its degradation. Because the demand for *M*. *dubia* continues to rise, selection and breeding can allow easier and more efficient cultivation, and thus prevent the destruction of the natural populations.

## Conclusion

This study found a low morphological variability within and among wild and cultivated populations of *M*. *dubia*. From all detected characteristics, only fruit parameters had the tendency to be different. However, those could be possibly explained by different environmental conditions in which the populations were grown, or by the lack of collected data. After evaluation of the fruits, it was found that the cultivated populations chosen for our study have not yet passed through any process of domestication, and had more-or-less the same morphological characteristics as the wild varieties.

Seven of the eight microsatellite loci developed were polymorphic and showed a high level of genetic diversity within and among populations. Bayesian analyses divided the wild populations into two main groups (a group from the river Putumayo and a group from the rivers Curaray, Tigre, Napo, and Itaya), and were able to show us the origin of cultivated populations. For this reason, microsatellite primers developed within this study can be recommended for further population-genetic studies of *M*. *dubia*.

Our research showed that populations located in different watersheds also possessed a different genetic composition. The origin of each population assigned according to each watershed increased the genetic distance between the populations and, thus, overall genetic diversity. Genetic diversity among and within cultivated populations was also relatively high, thanks to the different origin and generative propagation of plants by the use of seeds. Transfer of seeds and seedlings by local inhabitants plays a crucial role in the preservation of this genetic diversity.

This diversification could be used in the future breeding of this species. The crossbreeding of the individual trees from different geographical locations that suffer from inbreeding, and are genetically different, could result in a heterosis effect, and the resulting hybrids will possess higher genetic diversity, which might be more suitable for growing in plantations.

## Supporting information

S1 Fig Principal Coordinate Analysis (PCoA). (TIFF)

S2 Fig An analysis of similarity coefficients. (JPG)

S3 Fig Delta *K* graph (Δ*K*). (TIFF)

S1 Table Quantitative morphological descriptors for the wild and cultivated populations of camu-camu. Including the number of samples (**n**), mean, median, and standard deviation (**SD**) of each characteristic. Red-marked data with an asterisk were found to be statistically significant at probability level p = 0.05.
(DOC)

S2 Table Main coefficients of genetic diversity for wild and cultivated populations of camu-camu with two-sided p-values obtained after 10,000 permutations. **Ho**, observed heterozygosity; **He**, expected heterozygosity; **F**, fixation index; **Fst**, fixation index of a subpopulation relative to the total population.
(DOC)
