# Supplementary figures and images for: Morphological and genetic diversity of camu-camu [Myrciaria dubia (Kunth) McVaugh] in the Peruvian Amazon
Source: PLoS One. 2017 Jun 28;12(6):e0179886. doi: 10.1371/journal.pone.0179886 (PMC5489195; doi:10.1371/journal.pone.0179886)

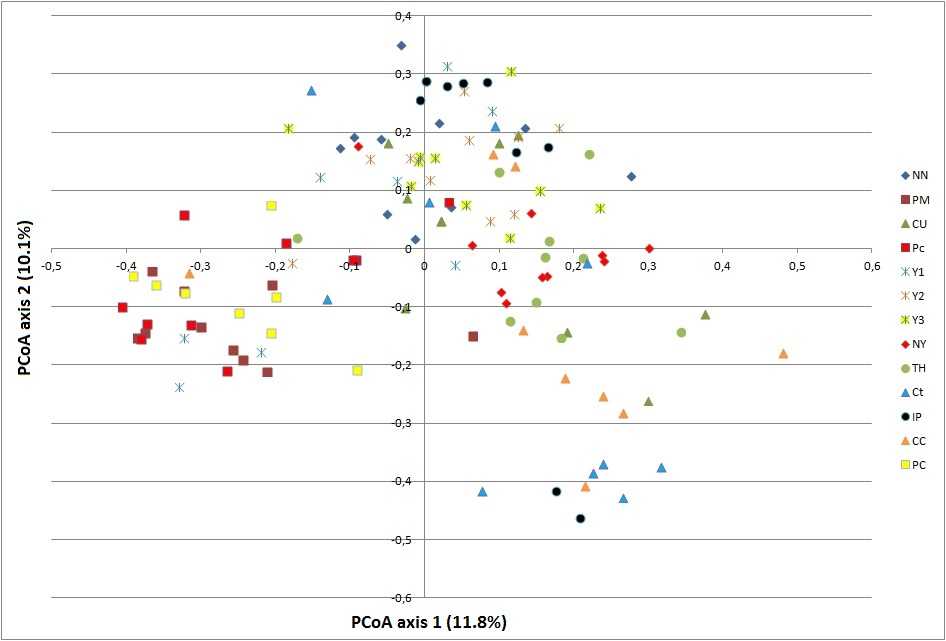

Supplement: S1 Fig — (TIFF) [file pone.0179886.s001.tiff]

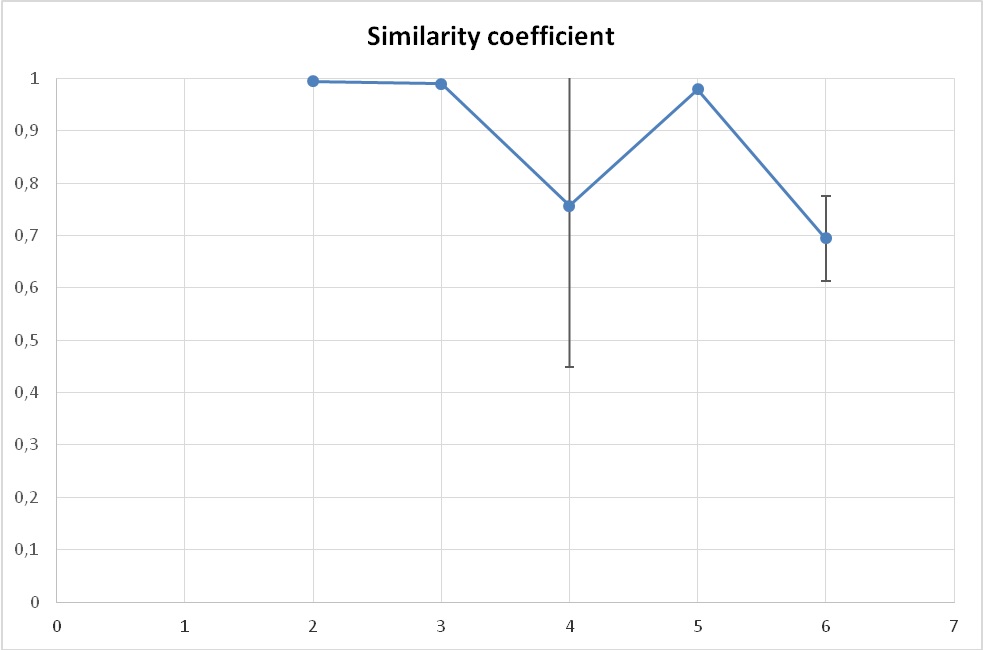

Supplement: S2 Fig — (JPG) [file pone.0179886.s002.jpg]

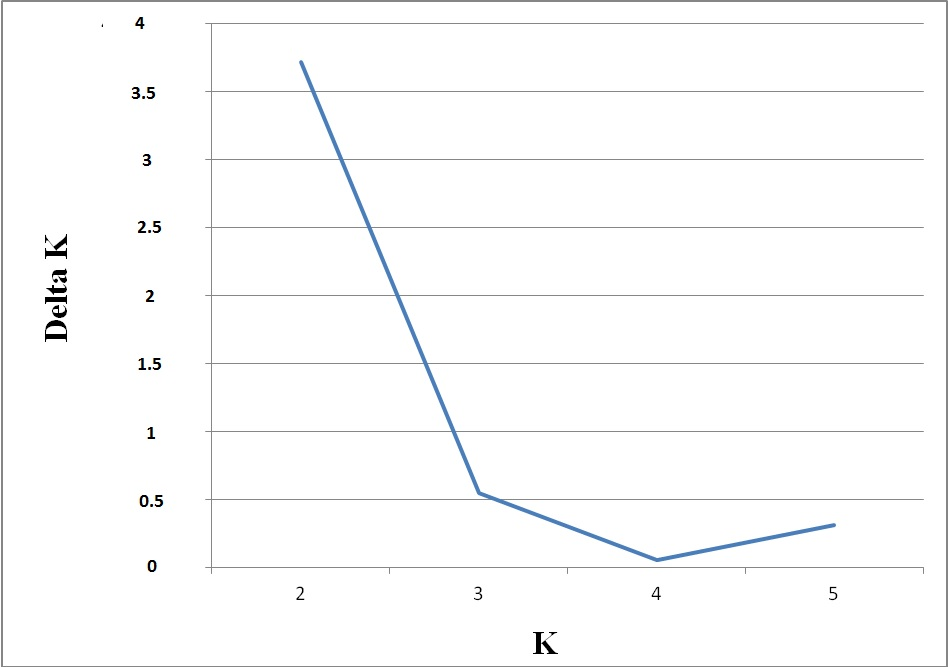

Supplement: S3 Fig — (TIFF) [file pone.0179886.s003.tiff]
