# Supplementary material for: Morphological and genetic diversity of camu-camu [Myrciaria dubia (Kunth) McVaugh] in the Peruvian Amazon
Source: PLoS One. 2017 Jun 28;12(6):e0179886. doi: 10.1371/journal.pone.0179886 (PMC5489195; doi:10.1371/journal.pone.0179886)
Supplement: S1 Table — Including the number of samples (n), mean, median, and standard deviation (SD) of each characteristic. Red-marked data with an asterisk were found to be statistically significant at probability level p = 0.05. (DOC) [file pone.0179886.s004.doc]

Table S1. Quantitative morphological descriptors for the wild and cultivated populations of camu-camu, including number of samples (**n**), mean, median and standard deviation (**SD**) of each characteristic. Red marked data with star were found to be statistically significant at probability level p=0.05

| Parameter | Wild population (W) | | | | Cultivated population (C) | | | |
| --- | --- | --- | --- | --- | --- | --- | --- | --- |
|  | N | mean | median | SD | N | mean | median | SD |
| leaf length | 50 | 9.14 | 9.21 | 0.95 | 15 | 9.05 | 9.07 | 0.79 |
| leaf width | 50 | 3.40 | 3.37 | 0.43 | 15 | 3.23 | 3.19 | 0.39 |
| length/width | 50 | 2.72 | 2.72* | 0.33 | 15 | 2.83 | 2.89* | 0.32 |
| petiole length | 50 | 0.70 | 0.63 | 0.22 | 15 | 0.82 | 0.67 | 0.42 |
| number of flowers | 17 | 4.07 | 4.00 | 1.21 | 14 | 3.66 | 3.57 | 1.16 |
| size of fruit | 9 | 2.60 | 2.59 | 0.24 | 14 | 2.54 | 2.54 | 0.19 |
| weight of fruit (g) | 9 | 11.22 | 10.82 | 2.70* | 14 | 9.82 | 9.75 | 1.74* |
| weight of seed (g) | 9 | 2.39 | 2.31 | 0.74 | 14 | 2.71 | 2.74 | 0.66 |
| weight of pulp (g) | 9 | 8.83 | 8.54 | 2.15* | 14 | 7.11 | 7.05 | 1.23* |
| number of seeds | 9 | 2.44* | 2.67 | 0.81 | 14 | 2.94* | 3.00 | 0.70 |

N- number of valid examples
