# Supplementary material for: Morphological and genetic diversity of camu-camu [Myrciaria dubia (Kunth) McVaugh] in the Peruvian Amazon
Source: PLoS One. 2017 Jun 28;12(6):e0179886. doi: 10.1371/journal.pone.0179886 (PMC5489195; doi:10.1371/journal.pone.0179886)
Supplement: S2 Table — Ho, observed heterozygosity; He, expected heterozygosity; F, fixation index; Fst, fixation index of a subpopulation relative to the total population. (DOC) [file pone.0179886.s005.doc]

|  | Allelic richness | Ho | He | Fis | Fst |
| --- | --- | --- | --- | --- | --- |
| Wild pop. | 2.965 | 0.347 | 0.516 | 0.328 | 0.170 |
| Cultivated pop. | 2.997 | 0.404 | 0.506 | 0.200 | 0.118 |
| p-values | 0.826 | 0.273 | 0.786 | 0.104 | 0.471 |

Table S2. Main coefficients of genetic diversity for wild and cultivated populations of camu-camu with two-sided p-values obtained after 10, 000 permutations (**Ho**: observed heterozygosity, **He**: expected heterozygosity, **Fis**: inbreeding coefficient, **Fst***:* fixation index of a subpopulation relative to the total population)
